# A Importância de Reconhecer a Co-ocorrência de Fatores de Risco Cardiometabólico na População para Estabelecer Prioridades em Políticas Públicas

**DOI:** 10.36660/abc.20210511

**Published:** 2021-07-15

**Authors:** Helena Cramer Veiga Rey

**Affiliations:** 1 Instituto Nacional de CardiologiaRio de JaneiroRJBrasilInstituto Nacional de Cardiologia, Rio de Janeiro, RJ - Brasil

**Keywords:** Síndrome X Metabólica, Morbimortalidade, Fatores de Risco, Proteína C-Reativa, Espessura Intima–Média Carotídea, Hipertensão, Obesidade, Diabetes Mellitus, Saúde Pública

A doença cardiometabólica (DCM) é a principal causa de morbimortalidade em todo o mundo.^1^ A síndrome metabólica (SM) é um conjunto de fatores de risco para doenças metabólicas, incluindo pressão arterial elevada, hipertensão, hiperglicemia, dislipidemia e obesidade. Quando esses fatores de risco estão presentes em combinação, a probabilidade de futuros problemas cardiovasculares aumenta mais do que quando qualquer um dos riscos está presente sozinho.^2^

A SM tem uma frequência estimada entre 20% e 25% na população adulta em todo o mundo, e a prevalência aumenta com a idade.^3;4^

Em um estudo transversal de base populacional com dados laboratoriais da Pesquisa Nacional de Saúde de 2014-2015, a prevalência da Síndrome Metabólica (SM) foi estimada em 38,4% na população brasileira.^5^ De acordo com a Pesquisa Nacional de Saúde, os subgrupos que são sociodemograficamente vulneráveis e têm estilos de vida pouco saudáveis têm uma prevalência maior de SM.^6;7^

Pesquisas anteriores estabeleceram associações entre indicadores inflamatórios, aterosclerose e componentes da SM.^8;9^ É fundamental entender a co-ocorrência de fatores de risco cardiometabólico e sua associação com inflamação crônica e doença aterosclerótica para controlar melhor os fatores de risco de uma maneira multifacetada.

Com base nisso, Lima et al.,^10^ tiveram como objetivo caracterizar grupos de fatores de risco cardiometabólico e sua relação com a aterosclerose e inflamação crônica em adultos e idosos residentes no sul do Brasil. Foi realizada uma análise transversal baseada em censo de dados de duas coortes populacionais de adultos e idosos (EpiFloripa Adult e Aging Cohost Studies) para determinar a associação entre variáveis como pressão arterial, circunferência da cintura, exames laboratoriais de perfil lipídico e de glicose, isoladas ou combinadas, com os resultados da espessura da camada íntima-média da carótida, placas ateroscleróticas e níveis séricos de proteína C-reativa.

O estudo mostrou que indivíduos com componentes da síndrome metabólica em grupos estavam relacionados com aumento da espessura da artéria carótida e níveis de proteína C-reativa em comparação com pessoas sem SM. O aumento da circunferência da cintura foi um preditor prevalente de inflamação, e o agrupamento de circunferência da cintura elevada com hipertensão arterial estava associado ao aumento dos níveis de aterosclerose e proteína C-reativa. A espessura da camada íntima-média e da proteína C-reativa associada aumentaram em proporção ao número de fatores de risco presentes no mesmo indivíduo. Os agrupamentos de fatores de risco para inflamação e aterosclerose incluíram obesidade central e hipertensão, ambos modificáveis.^10^

Os estudos de coorte que avaliam os fatores de risco cardiovascular são extremamente importantes para estabelecer as prioridades de saúde pública e o ponto vital é que se trata de um estudo de base populacional, onde os dados foram medidos e coletados através de um método apropriado, que incluiu adultos de renda média e idosos no Brasil, mas que não deveria ser extrapolado para diferentes populações.

A prevalência estimada de obesidade em adultos é de 23,5% no sul do Brasil^11^ e, de acordo com a pesquisa nacional de saúde, esse número dobrou nas últimas duas décadas.^12^ De acordo com a Pesquisa Nacional de Saúde, a prevalência de hipertensão em 2013 era de 22,8% e aumentou com a idade.^13^

Intervenções de base populacional para controle de peso e pressão arterial são urgentemente necessárias. Foi feita uma estimativa dos custos atribuíveis às doenças crônicas não transmissíveis a partir dos riscos relativos e prevalência populacional de hipertensão, diabetes e obesidade, considerando custos de internações, procedimentos ambulatoriais e medicamentos distribuídos pelo sistema público de saúde no Brasil (SUS) para tratar essas doenças na população adulta do Brasil. O custo do SUS atribuível à hipertensão foi de R$2,03 bilhões e à obesidade em 2018 foi de R$1,42 bilhão.^14^ Juntos, esses custos representam cerca de 4,2% do orçamento anual do sistema público de saúde no Brasil.

As principais recomendações para a prevenção e tratamento da SM são mudanças no estilo de vida com foco na educação, atividade física frequente e dieta nutritiva, bem como intervenções medicamentosas.^15,16^ Revisões sistemáticas de ensaios clínicos randomizados indicam que programas de mudança de estilo de vida têm benefícios no controle da SM e impacto na qualidade de vida.^15,17^

Em um país que possui um sistema de saúde universal, e a maior parte da população depende do financiamento público da saúde, saber onde alocar os recursos é fundamental para um melhor controle da morbimortalidade das DCM.

## References

[1] . World Health Organization. (WHO)_World health statistics 2018: monitoring health for the SDGs, sustainable development goals. 2018. Disponível em: < https://www.who.int/data/gho/data/themes/topics/topic-details/GHO/world-health-statistics >. Acesso em: 2nd July 2019.

[2] . Sposito A, Caramelli B, Fonseca FA, Bertolami MC, Afiune Neto A, Souza AD, et al.[IV Brazilian Guideline for Dyslipidemia and Atherosclerosis prevention: Department of Atherosclerosis of Brazilian Society of Cardiology]. Arq Bras Cardiol.2007;88(supl1):2-19. 10.1590/s0066-782x2007000700002 17515982

[3] . Alberti KG, Eckel R, Grundy SM, Zimmet PZ, Cleeman JI, Donato KA, et al.Harmonizing the metabolic syndrome: a joint interim statement of the International Diabetes Federation Task Force on Epidemiology and Prevention; National Heart, Lung, and Blood Institute; American Heart Association; World Heart Federation; International Atherosclerosis Society; and International Association for the Study of Obesity. Circulation. 2009;120(16):1640-5. 10.1161/CIRCULATIONAHA.109.192644 19805654

[4] . Saad M AN, Cardoso GP, Martins WA, Velarde LG, Cruz Filho RA. Prevalence of metabolic syndrome in elderly and agreement among four diagnostic criteria. Arq Bras Cardiol.2014;102(3):263-9. 10.5935/abc.20140013 PMC3987322 24676226

[5] . Oliveira LVA, Santos BNS, Machado IE, Malta DC, Velasquez-Nelendez G, Felisbino Mendes MS.Prevalence of the Metabolic Syndrome and its components in the Brazilian adult population. Cien Saude Colet.2020;25(11):4269-80. 10.1590/1413-812320202511.31202020 33175036

[6] . Ramires KN, Menezes RC, Longo-Silva G, Gama dos Santos T, Marinho PM, Silveira JAC. Prevalence and Factors Associated with Metabolic Syndrome among Brazilian Adult Population: National Health Survey - 2013. Arq Bras Cardiol. 2018;110(5):455-66. 10.5935/abc.20180072 PMC5967140 29898045

[7] . Francisco PMS, Assumpção D, Malta DC. Co-occurrence of Smoking and Unhealthy Diet in the Brazilian Adult Population. Arq Bras Cardiol.2019;113(4):699-709. 10.5935/abc.20190222 PMC7020877 31691752

[8] . Vu JD, Vu JB, Pio JR, Malik S, Franklin SS, Chen RS, et al.. Impact of C-reactive protein on the likelihood of peripheral arterial disease in United States adults with the metabolic syndrome, diabetes mellitus, and preexisting cardiovascular disease. Am J Cardiol.2005;96(5):655-8. 10.1016/j.amjcard.2005.04.038 16125489

[9] . Garcia VP, Rocha HN, sales AR, Rocha NG, Nobrega ACL. Sex Differences in High Sensitivity C-Reactive Protein in Subjects with Risk Factors of Metabolic Syndrome. Arq Bras Cardiol.2016;106(3):182-7. 10.5935/abc.20160027 PMC4811272 27027366

[10] . Lima TR, Silva DAS, Giehl MWC, D’Orsi E, González-Chica DA. Clusters of Cardiometabolic Risk Factors and Their Association with Atherosclerosis and Chronic Inflammation among Adults and Elderly in Florianópolis, Southern Brazil. Arq Bras Cardiol. 2021; 117(1):39-48. 10.36660/abc.20200230 PMC8294721 34320066

[11] . Vedana EH, Peres MA, Neves J, Rocha GC, Longo GZ. Prevalence of obesity and potential causal factors among adults in southern Brazil. Arq Bras Endocrinol Metabol.2008;52(7):1156-62. 10.1590/s0004-27302008000700012 19082304

[12] . Szwarcwald CL, Malta DC, Pereira CA, Vieira MLF, Conde WL, Souza Jr PRB, et al.. [National Health Survey in Brazil: design and methodology of application. Cien Saude Colet.2008;52(7):333-42. 10.1590/1413-81232014192.14072012 24863810

[13] . Malta DC, Santos NB, Perillo RO, Szwarcwald CL.Prevalence of high blood pressure measured in the Brazilian population, National Health Survey, 2013. São Paulo Med J.2016;134(2):163-70. 10.1590/1516-3180.2015.02090911 PMC10496535 27224281

[14] . Nilson EAF, Andrade RCS, Brito DA, Oliveira ML. Costs attributable to obesity, hypertension, and diabetes in the Unified Health System, Brazil, 2018Costos atribuibles a la obesidad, la hipertensión y la diabetes en el Sistema Único de Salud de Brasil, 2018. Rev Panam Salud Publica.2020;44:e32. 10.26633/RPSP.2020.32 PMC7147115 32284708

[15] . Saboya PP, Bodanese LC, Zimmerman PR, Gustavo AS, Macagnan FE, Zimmerman PR, ty al.Lifestyle Intervention on Metabolic Syndrome and its Impact on Quality of Life: A Randomized Controlled Trial. Arq Bras Cardiol.2017;108(1):60-9. 10.5935/abc.20160186 PMC5245849 27982160

[16] . Executive Summary of The Third Report of The National Cholesterol Education Program (NCEP) Expert Panel on Detection, Evaluation, And Treatment of High Blood Cholesterol In Adults (Adult Treatment Panel III). JAMA.2001;285(19):2486-97. 10.1001/jama.285.19.2486 11368702

[17] . Bassi N, Karogodin I, Wang S, Vassallo P, Priyanath A, Massaro E, et al. Lifestyle modification for metabolic syndrome: a systematic review. Am J Med. 2014;127(12):1242.e1-10. 10.1016/j.amjmed.2014.06.035 25004456

