# Supplementary material for: A Importância de Reconhecer a Co-ocorrência de Fatores de Risco Cardiometabólico na População para Estabelecer Prioridades em Políticas Públicas
Source: Arq Bras Cardiol. 2021 Jul 15;117(1):49–50. [Article in Portuguese] doi: 10.36660/abc.20210511 (PMC8294716; doi:10.36660/abc.20210511)
Supplement: Translation

# Translation: The Importance of Recognizing the Co-Occurrence of Cardiometabolic Risk Factors in the Population to Establish Priorities in Public Policies

**Authors:** Helena Cramer Veiga Rey

**Affiliations:** 1 BrazilInstituto Nacional de Cardiologia, Rio de Janeiro, RJ - Brazil

**Keywords:** Metabolic Syndrome X, Morbimortality, Risk Factors, C- Reactive Protein, Carotid Intima-Media Thickness, Hypertension, Obesity, Diabetes Mellitus, Public Health

Cardiometabolic disease (CMD) is the leading cause of morbidity and mortality around the globe.^1^ Metabolic syndrome (MS) is a collection of risk factors for metabolic disease, including elevated blood pressure, hypertension, hyperglycemia, dyslipidemia, and obesity. When these risk factors are present in combination, the likelihood of future cardiovascular issues increases more than when any of these risks is present alone.^2^

MS has an estimated frequency between 20% and 25% in the adult population worldwide, and the prevalence increases with age.^3;4^

In a cross-sectional population-based investigation using laboratory data from the 2014–2015 National Health Survey, the prevalence of Metabolic Syndrome (MS) was estimated to be 38.4% in the Brazilian population.^5^ According to the National Health Survey, subgroups that are sociodemographically vulnerable and have unhealthy lifestyles have a higher prevalence of MS.^6;7^

Previous research has established associations between inflammatory indicators, atherosclerosis, and MS components.^8;9^ It is critical to understand the co-occurrence of cardiometabolic risk factors and their association with chronic inflammation and atherosclerotic disease to better control risk factors in a multifaceted way.

Based on this, Lima et al.^10^ aimed to characterize clusters of cardiometabolic risk factors and their relationship with atherosclerosis and chronic inflammation in adults and the elderly living in southern Brazil. A census-based, cross-sectional analysis of data from two population cohorts of adults and elderly (EpiFloripa Adult and Aging Cohost Studies) was conducted to determine the association between variables such as blood pressure, waist circumference, laboratory tests of lipid and glucose profile, isolated or in combination, with the outcomes of carotid intima-media thickness, atherosclerotic plaques, and serum levels of – C-reactive protein.

The study showed that individuals with the metabolic syndrome components in groups were related with increased carotid artery thickness and C-reactive protein levels, as compared to individuals without MS. Increased waist circumference was a prevalent predictor of inflammation, and the clustering of high waist circumference with arterial hypertension was associated with increased atherosclerosis and C-reactive protein levels. The intima-media thickness and associated protein C increased in proportion to the number of risk factors present in the same individual. The clusters of risk factors for inflammation and atherosclerosis included central obesity and hypertension, which are both modifiable.^10^

Cohort studies that assess cardiovascular risk factors are extremely important to establish public health priorities and the vital point is that this is a population-based study, where data were measured and collected using an appropriate method, which included middle-income adults and older adults in Brazil but should not be extrapolated to different populations.

The estimated prevalence of obesity in adults is 23.5% in southern Brazil,^11^ and, according to the national health survey, this number has doubled in the last two decades.^12^ According to the National Health Survey, the prevalence of hypertension in 2013 was 22.8% and increased with age.^13^

Population-based interventions for weight and blood pressure control are urgently needed. An estimate was carried out of the costs attributable to non-communicable chronic diseases based on the relative risks and population prevalence of hypertension, diabetes, and obesity, considering costs of hospitalizations, outpatient procedures, and drugs distributed by the public health system in Brazil (SUS) to treat these diseases in the adult population in Brazil. The SUS cost attributable to hypertension was R$2.03 billion, and to obesity in 2018 was R$1.42 billion.^14^ These costs together make up about 4.2% of the annual budget of the public health system in Brazil.

The key recommendations for MS prevention and treatment are lifestyle changes focused on education, frequent physical activity, and a nutritious diet, as well as drug interventions.^15;16^ Systematic reviews of randomized clinical trials indicate that lifestyle change programs have benefits for the control of MS and an impact on quality of life.^15;17^

In a country that has a universal health system, and most of the population depends on public health financing, knowing where to allocate resources is crucial for better control of CMD morbidity and mortality.
